# Novel use of peak optimized particle therapy for the delivery of three-dimensional spatially fractionated radiotherapy

**DOI:** 10.3389/fonc.2026.1736260

**Published:** 2026-03-11

**Authors:** James W. Snider, Pouya Sabouri, Sina Mossahebi, Arpit M. Chhabra, Jason K. Molitoris, Tejan Diwanji, Katja M. Langen, William F. Regine, Mingyao Zhu, Charles B. Simone

**Affiliations:** 1 Partners in Healthcare Technology, LLC, Alpharetta, GA, United States; 2 Proton International, LLC, Alpharetta, GA, United States; 3 Department of Radiation Oncology, University of Maryland School of Medicine, Baltimore, MD, United States; 4 Department of Radiation Oncology, University of Arkansas for Medical Sciences, Little Rock, AR, United States; 5 New York Proton Center, New York, NY, United States; 6 Department of Radiation Oncology, Mid-Atlantic Permanente Medical Group PC, Rockville, MD, United States; 7 Department of Radiation Oncology, Emory University School of Medicine, Atlanta, GA, United States

**Keywords:** FLASH radiotherapy, lattice, particle therapy, proton therapy, spatially fractionated radiotherapy

## Abstract

Spatially fractionated radiotherapy (SFRT) has been delivered safely and effectively mostly utilizing beamlets of megavoltage photons in recent years. Particle therapy offers the promise of improved dose distributions with less exposure of surrounding normal tissues. However, most efforts have focused on mimicking the beamlets of traditional SFRT two-dimensional therapy (also known as GRID), potentially sacrificing advantages that could be achieved with scanned particle beams. We, herein, lay out the rationale for and execution of a novel pencil beam scanning proton therapy technique for SFRT that prioritizes pristine Bragg peak delivery in an interleaved and rotated three-dimensional SFRT (LATTICE) format. We propose that this patented (US11478665B2) technique optimizes particle SFRT therapy radiobiologic effect, minimizes normal tissue and superficial dose, and allows for alternative SFRT applications across a course of therapy. This technique lends itself to further exploration in delivery techniques, fractionation, and ultra-high dose rates.

## Introduction

### SFRT approaches in radiation therapy

Although previous radiotherapy approaches favored homogeneous dose distributions across tumor targets, heterogeneous techniques have made a resurgence with the advent of stereotactic ablative radiotherapy (SABR) (1). Spatially fractionated radiotherapy (SFRT) represents an extreme example of heterogeneity within the tumoral target that can yield dose distributions similar to a virtual form of brachytherapy (2). Invented in the early-1900s, and attributed to Alban Köhler, out of necessity to reduce high dose exposure to a confluent area of skin overlying a tumor, two-dimensional SFRT (GRID) was primarily used for this purpose with low-energy therapeutic beams (3). With the advent of orthovoltage beams, deep-seated tumors could be approached with reduced skin and subcutaneous toxicity by spatially separating the high-dose regions with a collimator to spare skin between the “beamlets” (4). In the megavoltage (MV) era, GRID has been repurposed to deliver streaks of high doses separated by areas of low-to-moderate doses, especially as additional dose escalation to conventionally fractionated courses or as palliation in bulky tumors. Substantial tumor responses and shrinkage have been documented with this approach using MV-energy linear accelerators (5–8).

Despite the availability of three-dimensional (3D) imaging and planning, most therapeutic SFRT delivered in the modern era has primarily used a two-dimensional technique and approach, achieved by either traditional collimator or multileaf collimator (MLC) employment (5–8). This approach comes with significant limitations, primarily from the characteristic pattern of dose deposition with MV photon beams in tissue. Most therapeutic MV beams available on modern linear accelerators possess a single dose maximum (Dmax) peak that is 1.5 (6 MV)–3.2 (18 MV) cm deep in tissue. When employing a single photon beam with either a collimator or MLC GRID approach, this imparts a dose peak that lies shallowly subcutaneous, followed by a rapid dose falloff streak that carries through the remainder of the target. With GRID doses ranging generally from 12 to 20 Gy, prescribed to Dmax in a single fraction, this technique can also expose normal tissues proximal or distal to the target to substantial doses that may result in toxicity.

As a solution, three-dimensional-high dose LATTICE radiotherapy was developed and has been utilized with intensity-modulated photon radiation therapy techniques (9). LATTICE leverages intensity-modulated radiation therapy and inverse planning to deliver islands of high dose that are three-dimensionally (rather than two-dimensionally) distributed within a target. Static beam and rotational techniques (volumetric-modulated arc therapy or tomotherapy) have been shown to be able to achieve these distributions (10).

Proton therapy offers promise of further improved dose distributions in delivery of GRID radiotherapy. Proton therapy maintains several advantages over photon therapy for this application, as discussed in this article. However, the primary advantage stems from the proton beam’s limited range, which can be controlled based on the energy of the incident beam (11, 12). With pencil beam scanning techniques, desired dose distribution may be obtained without collimators and with greater spatial freedom in placement of each dose peak/valley. To fully appreciate the myriad opportunities available with proton-based SFRT, a review is necessary of the radiobiologic rationale for SFRT radiotherapy and its advantages in an overall treatment strategy. Following that, a novel, now patented (US11478665B2) approach that leverages synergies between SFRT and pencil beam scanning proton therapy will be described in detail. Finally, future areas for exploration with this technique will be proposed.

### Radiobiologic rationale for SFRT

Hypofractionated radiotherapy, which delivers high doses per fraction, has been shown to be quite effective when treating relatively small targets in the stereotactic radiosurgery and SABR settings (13, 14). In these techniques, fraction sizes regularly exceed 10 Gy, and the total dose is delivered over 1–2 weeks. Better than expected (per α/β ratio and linear quadratic model) disease control has resulted with these techniques (15). Despite the promise suggested by this approach, however, its applicability to large tumors is limited by overexposure of nearby normal tissues. SFRT can allow for use of the radiobiologic advantage associated with high dose per fraction deliveries without “overdosing” neighboring critical structures. SFRT, by its nature, incorporates valleys of low dose that are distributed throughout the target tumor, but that can also be preferentially favored towards organs-at-risk as needed. It should be noted, however, that the *in toto* radiobiologic effects of SABR and SFRT are intended to substantially differ based on the high dose complete coverage of tumor in SABR and the intentional dose valleys in SFRT. High dose per fraction to large tumors is not the sole advantage of SFRT, and numerous other mechanisms of radiobiologic effect have been proposed.

In clinical applications of SFRT, an important phenomenon has been observed: although GRID distribution usually conveys a highly heterogeneous dose, uniform tumoral regression is usually observed (16, 17). In attempting to clarify the semantics surrounding such effects, Blyth and Sykes have developed a categorization, identifying three mechanisms: abscopal, bystander, and cohort effects (18). Abscopal effects have rarely been documented in the radiotherapy literature but include tumoral response in a metastatic lesion distant from the target tumor being irradiated. In recent years, with the increased utilization of immunotherapy, growing interest has focused on revisiting this effect, but data are limited on ways to activate it. Numerous presentations have suggested that abscopal responses may be immunologically based and best elicited with high dose-per-fraction approaches (19). Serological markers of immune activation in the setting of SABR seem to confirm this (20–23). Also, delivering very low doses to the nontarget tumor seems to augment the abscopal effect (19). SFRT is particularly suited to delivering very high doses (peaks) to parts of a tumor and much lower doses (valleys) to others. If abscopal effects are possible, or if they can be employed both in metastatic or likely subclinical metastatic (e.g. bulky primary tumors) settings, SFRT is an excellent tool for eliciting such a response despite larger primary tumors otherwise ineligible for high-dose, ablative dose approaches.

The bystander effect describes the cell signaling induction stimulated by areas of high dose in SFRT that affect nearby cells in the low-dose region. Such bystander factors/cytokines, such as tumor necrosis factor (TNF)-α, TNF-related apoptosis-inducing ligand, and ceramide, have each been shown to be induced by SFRT approaches (24–26). These factors are subsequently involved in initiating the cell death cascade. Both *in vitro* and *in vivo* models have confirmed the cellular stress, DNA damage, and cell death that are outside of the irradiated regions but effected by SFRT techniques (27). Finally, cohort effects describe more direct cell-to-cell communications, often through gap junctions, that cause cell death within an irradiated volume (28). This pathway is more relevant among subpopulations where the majority of cells are exposed to dose.

Outside of these explanations, SFRT data to date has also suggested increase overall tumor oxygenation, an effect that can persist for a substantial portion of any planned subsequent conventionally fractionated course (29). In addition, within the particularly high dose peaks (>10 Gy) of SFRT, endothelial cell apoptosis and vascular compromise have been observed (30–34). Increased overall oxygenation would lead to increased radiosensitivity in viable tumor cells, and vascular compromise adds another dimension of cell killing in peak regions.

In summary, SFRT affects the tumor microenvironment in ways unique to conventional radiotherapy, and this approach causes tumoral cell death and response through multiple mechanisms. Each of these is either equally triggered or enhanced by utilization of proton therapy for delivery.

### Proton/particle therapy for GRID/LATTICE delivery

Proton (or any charged particle) therapy lends several distinct advantages for delivery of GRID/LATTICE radiotherapy over photon approaches. Of note, the remainder of this discussion will primarily reference proton therapy, but, in point of fact, any particle therapy with limited range (carbon, helium, electron, etc.) or enhanced region of effect (e.g. neutron) could be employed in this technique.

First, the limited range of the beam and its unique dose deposition pattern (Bragg peak) allow for maximum dose delivery in the planned peaks with minimal dose to surrounding critical structures. Especially with pencil beam scanning delivery techniques, these peaks can be arranged freely throughout a tumoral target with optimal control of spacing, depth, and intensity. This allows for greater ability to manipulate the spatial distribution of the planned peaks and valleys throughout the target.

In addition, in recent years, it has been increasingly recognized that the radiobiologic effectiveness (RBE) of proton beams does not appear to be constant across the entirety of the spread-out Bragg peak (SOBP) usually used for homogeneous dose deposition in clinical proton delivery (35). In clinical practice and in all currently available planning software, an RBE correction of 1.1 has been uniformly employed for physical dose calculation to relate proton dose to that of photon beams. Instead, there seems to be substantially enhanced RBE at the “end of range” or at the distal edge of each individual proton beam’s deposition (36). This is generally considered a weakness of proton therapy, as the potentially radiobiologically enhanced, distal edge of a beam is generally planned to a region beyond the target tumor to allow for setup and range uncertainties. Therefore, concerns for higher than expected radiobiologic effect in surrounding normal tissues must be addressed. However, in spatially fractionated therapy, this could clearly represent a distinct advantage—further focusing antitumoral effect—as long as all peaks are planned within the tumoral volume. Associated higher immune activation from high dose per fraction SFRT with protons could also be a strategy for employment. Targeting viable, or hypoxic, or select tumoral subpopulations through positioning of the SFRT proton peaks could prove further enhancing of SFRT effects. Additionally, the potential arises for variation of the peak to valley distribution as multiple SFRT fractions during a course of treatment could be delivered; although not unique to proton-based SFRT, this approach may be especially feasible with protons in the setting of nearby dose-limiting tissues.

Some pencil beam scanning proton therapy devices, further, possess the ability to deliver ultra-high dose rates. Particularly high, especially over 100 Gy/s, dose rates (FLASH) have been theorized to protect more normal tissue while enhancing or maintaining anti-tumoral effects (37). Recent clinical trials have evinced the safety of this technique, while forthcoming data will be needed to verify its effectiveness and advantages (38). Pencil beam scanning proton therapy SFRT would easily achieve very high and FLASH dose rates to each Bragg peak (and traversed normal tissues in the entrance plateau) if delivered without a spread out Bragg peak (SOBP). If FLASH is not uniquely radiobiologically beneficial in this setting, at the very least, the speed of delivery would allay many concerns regarding delivery of this approach in mobile targets – interplay effect mitigation (39).

The potential for proton therapy in the delivery of SFRT has not gone unrecognized. In fact, several groups have previously suggested its use or initiated it in clinical practice (40–42). None of the described approaches, however, have employed this technique in an arguably ideal fashion. The described techniques have varied widely. Currently employed approaches broadly include:

- Employment of SOBPs that are spatially fractionated in two dimensions to generate high-dose streaks through the target tumor much like beamlet distribution in GRID techniques but with a more consistent high-dose region along the path of the beamlet – mimicking more effectively a brachytherapy catheter dose distribution (43).
- Employment of stacked Bragg peaks and semi-SOBPs that are spatially fractionated in three dimensions with peaks separated along each beamlet path and perpendicular to the beamlet path (in the two other dimensions), much like a LATTICE distribution (44).
- Interleaved GRID beamlets or mini-GRID beamlets to achieve a homogeneous dose across the target while reducing proximal tissue toxicity by spatially fractionating entrance beamlets in two dimensions (42).

The last of these employs SFRT individual beam techniques but is inconsistent with the central tenets of SFRT in that it generates an approximately homogeneous target dose distribution. It is, therefore, not intended for the same clinical scenarios/use and is outside the scope of this review. The former two will be contrasted with a proposed alternative technique.

### Peak optimized particle SFRT

**Figure 1:**
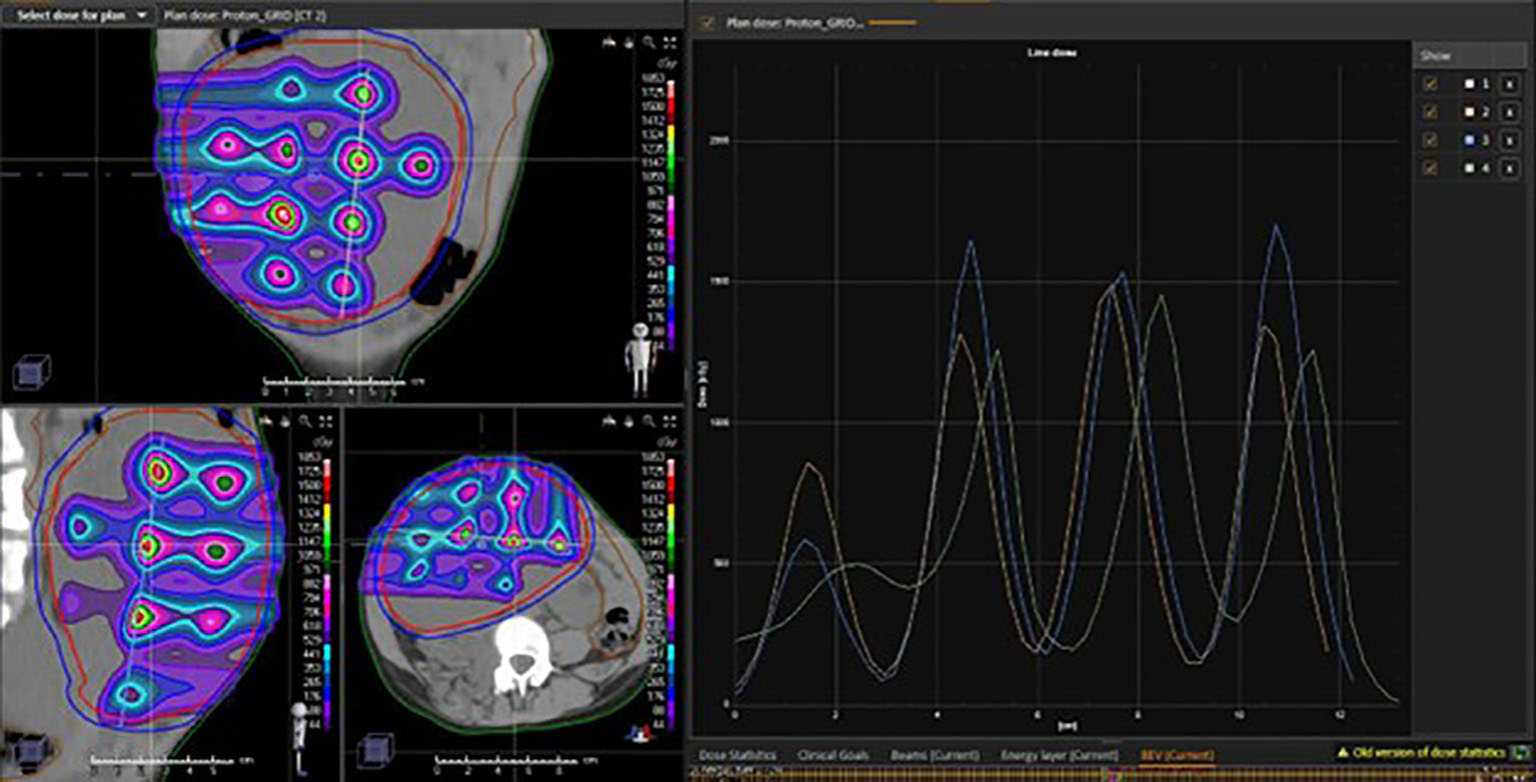
Example 2-beam pencil beam scanning proton therapy POP-SFRT technique with physical dose distribution and peak-valley dose line profile.

At the Maryland Proton Treatment Center, affiliated with the University of Maryland School of Medicine, we developed and planned an alternative method for SFRT delivery using pencil beam scanning proton therapy. This Peak Optimized Particle SFRT (POP-SFRT) technique prioritizes the use of “pristine Bragg peaks” by rarely, if ever, “stacking” peaks along the same beamlet path (**Figure 1**). In other words, each peak should be delivered along a unique (or nearly unique, in the event of unavoidable stacking) beamlet path. This approach generally requires that multiple beams be utilized to prevent overlap of entrance paths. The technique was described and patented by several of the current authors (US11478665B2). The primary aims of the POP-SFRT technique are to focus RBE-enhanced proton Bragg peaks within the target; lower proximal and distal normal tissue exposures versus current photon and proton SFRT approaches; and permit unique integrations into an overall treatment plan with multiple-SFRT fraction courses.

By planning in this manner, SOBPs are not utilized. This lends several key advantages over the two techniques described in (A) and (B). First, the entrance/superficial dose is substantially reduced. Proton therapy is generally not skin sparing because it lacks the build-up effect present in MV photon beams. If a confluent beamlet, as described in (A), is employed, this SOBP will necessitate delivery of almost the full dose at the skin and to all proximal intervening tissues before the beam reaches the target tissue. Option (B) offers some reduction in this regard, but almost completely limiting the stacking of Bragg peaks would further reduce superficial dose.

Second, by stacking Bragg peaks, techniques (A) and (B) would deliver pristine peaks at the distal edge of the tumor only, and all proximal peaks would necessarily be “built” by the addition of a new Bragg peak with the entrance plateau of any more distal Bragg peaks. This would likely lessen the enhanced RBE effect that accompanies use of the end of range of the proton beam in each SFRT peak. The POP-SFRT technique, by utilizing only pristine Bragg peaks for each SFRT peak, would optimally enhance RBE within each peak target. By utilizing lateral penumbra and dose falloff to fill in the valleys of the GRID distribution, the POP-SFRT technique would allow for greater degrees of freedom in peak spacing and peak-to-valley ratios.

Finally, by reducing dose to skin/proximal tissues and by improving planner control of peak-to-valley ratios, POP-SFRT would allow for optimization of peak placement to limit dose outside the target tumor similar to conventional fractionation (2–4 Gy) despite achieving peaks of 15–20 Gy. This may allow for delivery of the proton SFRT dose safely multiple times as an integrated boost to conventional fractionation rather than just once at the beginning or end of a conventionally fractionated radiotherapy course (45). Alternatively, completely POP-SFRT courses of radiotherapy could be developed, and regardless of the fractionation approach, the peaks could be intentionally varied in position, intensity, and angle of approach. This intentional variation in peak position promises opportunities to address tumoral subpopulations or regions differentially throughout a course of treatment; the logical translation of this re-placement of peaks towards adaptive courses of proton treatment is clear. Finally, adaptive courses of varied POP-SFRT should prove relatively easier to execute to traditional adaptive proton approaches as multiple spot maps could be pre-designed and selected by the treating team based on tumoral response, change, or uncertainties.

POP-SFRT plans are currently partially forward-planned in the absence of vendor-provided treatment planning software that address SFRT or especially POP-SFRT. These plans are achievable on generally any pencil beam scanning proton system with appropriate angles for patient approach.

**Figure 2:**
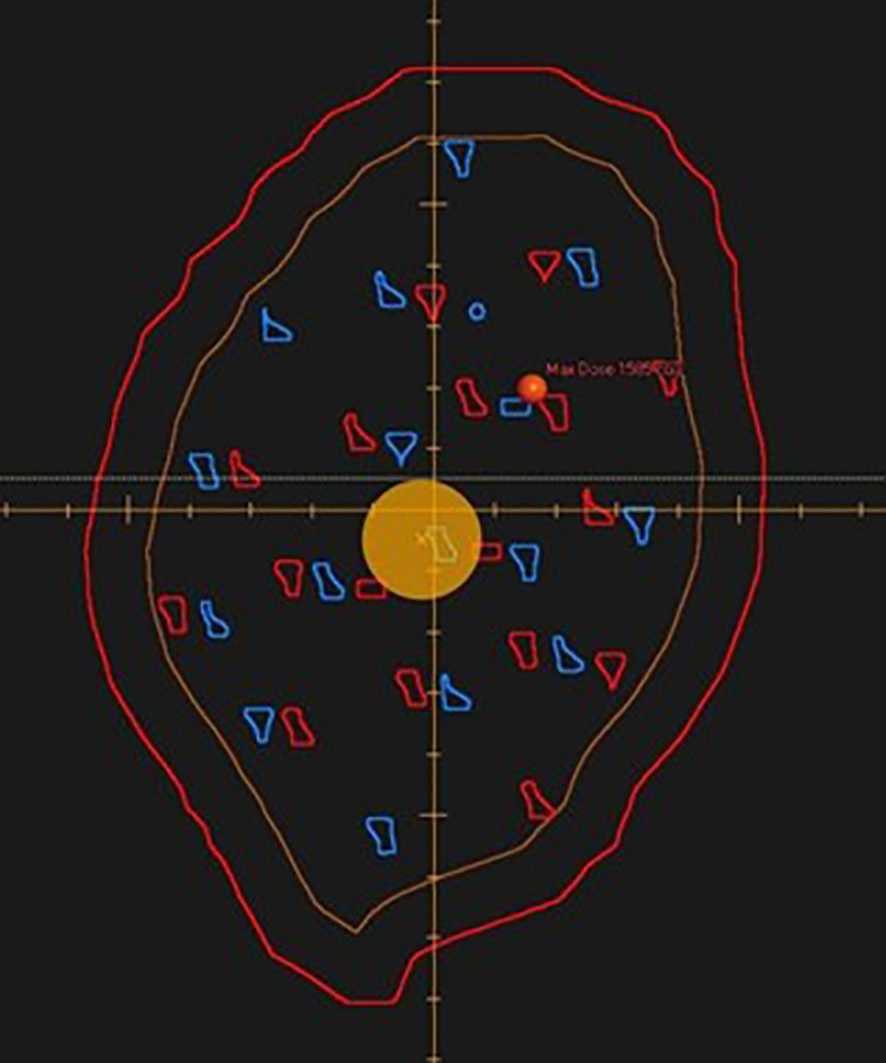
A beam’s eye view of POP-SFRT targets is displayed. The gross tumor volume is labeled in brown. The planning target volume for simultaneous integrated boost (low dose of 2 Gy) is labeled in dark red. POP-SFRT targets for beam 1 (blue) and POP-SFRT targets for beam 2 (red) are shown.

**Figure 3:**
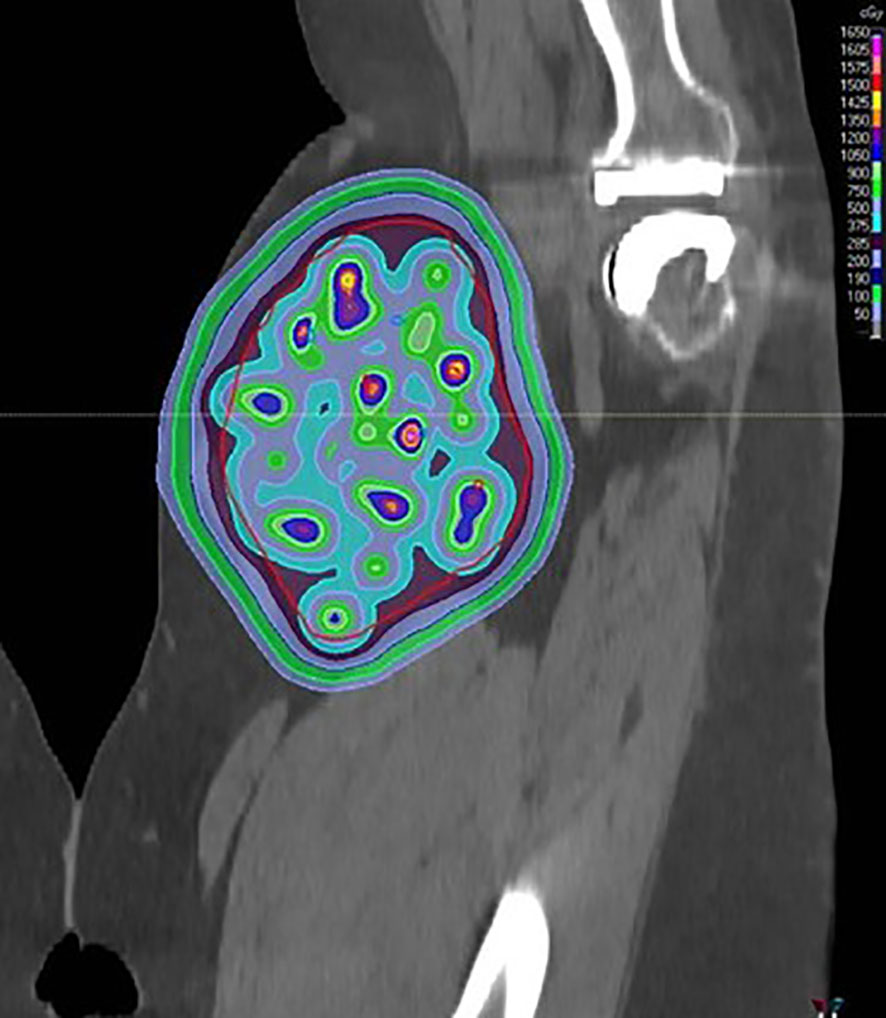
An example simultaneous integrated boost technique POP-SFRT ~15 Gy(RBE) peaks is shown in the coronal plane.

Although, many approaches may achieve similar results, the following is the reasonable first efforts to achieve the POP-SFRT effect with any pencil beam scanning proton planning software: Small subtargets (generally ~3-mm spheres) are placed within the target tumor at regular intervals in a “cascading” fashion by beams-eye-view of each beam employed (**Figure 2**) to prevent repeat use of the same beamlet path for delivery of two peaks (46). Optimization parameters are set to achieve peak doses in these small targets. Spot positions are manually deleted when outside of these subtargets. Most plans have required two to three beams to achieve this distribution without stacking Bragg peaks. A treatment planning script has been generated to automate placement of these targets and appropriate optimization parameters. Example plans have been generated both to employ POP-SFRT as a standalone technique/delivery as well as to achieve a simultaneous integrated boost on a background of a conventional 2-Gy fraction (**Figure 3**). A beam’s eye view of standalone POP-SFRT targets and a simultaneous integrated boost dose distribution are displayed in **Figures 2** and **3**, respectively. Peak-to-valley gradients have been modulated *in silico*, thus far, to approximately produce the 4:1 ratio recommended/achieved with 2D photon-based techniques with collimators. The mean dose across the target can be controlled primarily based on peak spacing and peak maximum doses. Peak spacing is generally on the order of multiple centimeters between peaks, although clarity of the ideal radiobiologic peak-to-valley ratio is still lacking. This distance, however, places peaks well outside of range and setup uncertainties for most disease sites – thus effectively preventing peak overlap. The resulting POP-SFRT dose distribution is highly heterogeneous, highly modular, and highly radiobiologically leveraged for the SFRT effect.

### Future directions for POP-SFRT

The proposed technique could be further tested or optimized in numerous ways. **Table 1** includes specific additions/investigations that have been suggested or planned. As previously mentioned, ultra-high dose rates or FLASH radiotherapy could be very easily achieved with such plans. These plans could possess improved normal tissue sparing while yielding at least equi-effective tumoricidal doses. Furthermore, very low dose rates or pulsed low dose rates could also be achieved if clinically advantageous.

**Table 1:** Future Possible Development for POP-SFRT.

Development area	Potential applications
Planning techniques	Inverse optimization algorithm for peak/valley placement and beamlet path selection. Robust inverse optimization for peak/valley placement and beamlet path selection. RBE and linear energy transfer–based planning application to this approach.
Device/technology pairing	Delivery with any limited range particle beam could be employed. Delivery with passive scattering systems could be achieved with template collimators/apertures and custom patient-specific compensators, or Collimators/MLCs could be utilized in the setting of pencil beam scanning to select beamlet size and sharpen penumbra. Minibeam techniques could be utilized with similar principles. Increased beam number could allow for further peak “packing” within the target; the extreme example could be proton arc–based techniques for delivery. Delivery with ultra-high or low dose rates are both possible.
Subtarget optimization	With delivery of multiple POP-SFRT fractions throughout a course of radiotherapy, peak placement could be altered and the solutions inversely optimized to be complementary in coverage. Peaks could be targeted to regions of solid tumor (rather than necrotic regions) based on imaging characteristics. Peaks could be targeted to regions of intratumoral hypoxia based on imaging characteristics. Peaks could be targeted to regions critical for tumoral perfusion based on imaging characteristics.
Delivery/therapeutic optimization	POP-SFRT could be utilized once in a treatment course or embedded with/replacing multiple fractions. POP-SFRT could be employed based on tumoral response or surrogate marker clearance to conventional course. POP-SFRT could be tested in multiple timings across therapy: beginning, midtreatment, end. POP-SFRT optimal peak/spot size/dose could be tested. POP-SFRT optimal peak-to-valley ratio could be tested. POP-SFRT optimal dose rate for delivery could be tested. Potential for ultra-high dose rate (FLASH) delivery. POP-SFRT pairing with additional therapies/modalities (e.g., surgical resection, immunotherapy [checkpoint inhibition], hyperthermia, etc.).

Particle arc therapy techniques are particularly well suited to achieving the pristine Bragg peaks required for this approach, while minimizing or eliminating entrance plateau overlaps that would create SOBPs. Whether dynamic arc deliveries or step and shoot, either could effectively deliver POP-SFRT. Multi-fraction approaches with varied peak placement could be explored with POP-SFRT.

In essence, the differing radiobiologic features of SFRT to conventional and even SABR approaches could be particularly enhanced and leveraged by this novel, POP-SFRT technique. Clinical investigation would be required to extensively test particular strengths and weaknesses of the technique in clinical applications and various disease entities.

## Conclusions

We report, herein, the general methodology and approach of POP-SFRT – a unique technique for delivery of proton-based spatially fractionated radiotherapy that has been achieved and subsequently patented. POP-SFRT promises potential radiobiologic advantages, improved normal tissue sparing, and provides alternative spatial fractionation applications over clinically active SFRT approaches. Further investigation and clinical trial development are warranted.

## Data Availability

The original contributions presented in the study are included in the article/supplementary material. Further inquiries can be directed to the corresponding author.
